# Aromatase Inhibitor-Induced Carpal Tunnel Syndrome Immunohistochemical Analysis and Clinical Evaluation: An Observational, Cross-Sectional, Case–Control Study

**DOI:** 10.3390/jcm14155513

**Published:** 2025-08-05

**Authors:** Iakov Molayem, Lucian Lior Marcovici, Roberto Gradini, Massimiliano Mancini, Silvia Taccogna, Alessia Pagnotta

**Affiliations:** 1 Hand Surgery and Microsurgery Unit, Jewish Hospital, 00100 Rome, Italy; i.molayem@gmail.com (I.M.); lucian.marcovici@gmail.com (L.L.M.); 2 Experimental Medicine Department, Sapienza University, 00100 Rome, Italy; roberto.gradini@uniroma1.it; 3 Morphologic and Molecular Pathology Unit, Sant’Andrea University Hospital, 00100 Rome, Italy; mamancini@ospedalesantandrea.it; 4 Pathology Unit, San Carlo di Nancy Hospital, 00100 Rome, Italy; staccogna@gvmnet.it

**Keywords:** estrogen, receptor, carpal tunnel syndrome, breast cancer, aromatase inhibitor

## Abstract

**Background/Objectives**: Breast cancer was the leading cause of malignant tumors among women in 2022. About two-thirds of breast cancer cases are hormone-receptor-positive. In these patients, aromatase inhibitors are a mainstay of treatment, but associated musculoskeletal symptoms can negatively affect patient compliance. Aromatase-inhibitor-induced carpal tunnel syndrome represents one of the main causes of aromatase inhibitor discontinuation, with a non-compliance rate of up to 67%, potentially leading to increased cancer mortality. This study investigates estrogen receptor expression in aromatase-inhibitor-induced carpal tunnel syndrome tissues, in order to better define its etiopathogenesis and derive preventive or therapeutic measures that can improve aromatase inhibitor patient compliance. To our knowledge, there is no study on this subject in the literature. **Methods**: Between 2023 and 2024, we recruited 14 patients at the Jewish Hospital of Rome, including seven patients with aromatase-inhibitor-induced carpal tunnel syndrome (study group) and seven with postmenopausal idiopathic carpal tunnel syndrome (control group). Each patient was evaluated based on a clinical visit, a questionnaire, instrumental exams, and serum hormone dosages and were treated with open carpal tunnel release surgery, during which transverse carpal ligament and flexor tenosynovium samples were collected. For immunohistochemical experiments, sections were treated with anti-estrogen receptor α and anti-estrogen receptor β antibodies. **Results**: The immunohistochemical features in the study and control groups were similar, demonstrating that tissues affected by aromatase-inhibitor-induced carpal tunnel syndrome are targets of direct estrogen action and that estrogen deprivation is correlated with disease etiogenesis. Surgery was effective in patient treatment. **Conclusions**: Aromatase-inhibitor-induced carpal tunnel syndrome represents a newly defined form of the disease. This syndrome represents one of the main causes of aromatase inhibitor discontinuation, due to its negative impact on the patient’s quality of life. The identification by clinicians of aromatase inhibitor use as a possible risk factor for carpal tunnel syndrome development is of essential importance, as early diagnosis and prompt management can improve patient compliance and overall breast cancer treatment outcomes.

## 1. Introduction

Breast cancer (BC) was the leading cause of malignant tumors in women in 2022, with 2.3 million new cases and 665,000 deaths. Among women, breast cancer accounts for one in four cancer cases and one in seven cancer deaths [1]. About two-thirds of BCs are hormone receptor (HR)-positive: aromatase inhibitors (AIs) are a mainstay of treatment [2], but associated musculoskeletal symptoms can negatively affect patient compliance.

Carpal tunnel syndrome (CTS) is the most prevalent chronic peripheral nerve compression syndrome [3]. This syndrome results from compression of the median nerve in the carpal tunnel, leading to sensory and motor disorders. Some of the risk factors for CTS suggest a hormonal role in this disease’s etiopathogenesis, including the use of AIs [4].

Aromatase-inhibitor-induced carpal tunnel syndrome (AI-CTS) represents a newly defined form of the disease. The etiopathogenesis of AI-CTS is not understood, but is likely related to estrogen deprivation. The prevalence of this form ranges from 0.8 [5] to 3.4% [6,7]. However, these values are likely underestimated due to the progressive increase in AI use and the lack of recognition of the disease.

AI-CTS represents one of the main causes of AI discontinuation, with a non-compliance rate of up to 67% [5], potentially leading to increased cancer mortality [8].

The aim of this study is to investigate estrogen receptor (ER) expression in AI-CTS tissues, in order to better define its etiopathogenesis and derive preventive or therapeutic measures that can improve AI patient compliance.

To our knowledge, there is no study on this subject in the literature.

## 2. Materials and Methods

This observational, cross-sectional, case–control prospective study was approved by the Jewish Hospital Review Board (protocol code 0000954; date of approval 23 June 2023; Rome, Italy), and the informed consent was obtained from all patients.

The study group (AI-CTS) inclusion criteria were as follows: women aged 50–60 years, with postmenopausal BC, AI treatment, CTS diagnosis, CTS symptom onset within 1 year of AI administration, and a lack of response to nonsurgical treatment.

The control group (postmenopausal idiopathic CTS) inclusion criteria were as follows: women aged 50–60 years, in menopause, diagnosed with CTS, CTS symptom onset within 1 year of menopause, and a lack of response to nonsurgical treatment.

Patients with CTS symptoms pre-existing AI administration or menopause, or any concomitant local or systemic disease related to CTS secondary forms, were excluded from the study.

To confirm the relation between symptom onset and AI treatment, a 2-week drug discontinuation test was performed in each patient in the study group, with immediate relief of symptoms. Re-exacerbation occurred as soon as AI was reintroduced. Moreover, to reduce a possible confounding factor, no patient with a symptom onset-to-menopause interval of < 1 year was enrolled in the study group.

At the Jewish Hospital (Rome, Italy), we recruited 14 patients between 2023 and 2024 (study group 7, control group 7); the minimum follow-up was set at 6 months.

Preoperatively, each case was evaluated through a clinical visit (history and physical examination), questionnaire (Italian version of Boston Carpal Tunnel Syndrome Questionnaire [9]), instrumental examinations (nerve conduction study, plain radiograph and ultrasound), and serum hormone dosages (estrone and 17βestradiol).

All patients underwent open carpal tunnel release surgery, during which transverse carpal ligament (TCL) and flexor tenosynovium (FT) samples were collected.

At 6 months of follow-up, each case was re-evaluated with a clinical visit and questionnaire. The resulting data are summarized in Table 1.

### 2.1. Surgical Procedure (Figure 1)

An experienced hand surgeon performed all surgical procedures.

A longitudinal skin incision was made in line with the fourth digit radial border, beginning at the proximal flexion crease and ending at the Kaplan cardinal line.

After TCL sectioning, the macroscopic appearance of the TCL, FT, and median nerve was detected and TCL, and FT samples were collected for microscopic evaluation. Finally, median neurolysis and flexor tenosynoviectomy were performed to complete the treatment.

### 2.2. Immunohistochemical Analysis

An experienced pathologist independently and blindly evaluated all specimens using a light microscope to ensure unbiased assessment.

Tissue samples were fixed in buffered formalin and subsequently embedded in paraffin. Serial sections, each 4 μm thick, were cut and prepared for immunohistochemical analysis.

Immunostaining was performed using an automated immunohistochemistry stainer. The following primary antibodies were applied:

- Mouse monoclonal anti-ERα (sc-8002, Santa Cruz Biotechnology, Santa Cruz, CA, USA, dilution: 1:25, incubation time: 2 h);
- Rabbit polyclonal anti-ERβ (ab5786, Abcam, Cambridge, UK, dilution: 1:300, incubation time: 30 min).

To ensure assay validity, control sections from the breast tissue were included in each staining run. Positive control sections were treated with the respective primary antibodies, while negative control sections underwent the same staining protocol but with the omission of primary antibodies. Both controls were processed under identical conditions to verify staining specificity and rule out nonspecific background signals.

Following immunostaining, whole-slide images (WSIs) were acquired using a high- resolution digital slide scanner, ensuring standardized imaging across all samples. These images were then analyzed using the QuPath Open Software for Bioimage Analysis (version 0.5.1, open source) for automated immunohistochemical evaluation.

The quantification of ERα and ERβ immunoreactivity was performed automatically through color thresholding-based segmentation, which allows for the precise detection and measurement of staining intensity and distribution across the entire tissue section. This process included the following steps.

1. Image Preprocessing: WSIs were normalized to ensure consistent background illumination and staining intensity across all samples.
2. Tissue Segmentation: Non-tissue areas, artifacts, and backgrounds were automatically excluded using QuPath’s region-of-interest (ROI) selection and thresholding functions.
3. Color Deconvolution: The hematoxylin and immunostain signals were separated using QuPath’s color deconvolution algorithm, isolating the diaminobenzidine (DAB)-positive staining corresponding to nuclear ERα and ERβ expression.
4. Thresholding and Classification: A customized color threshold was applied to detect and quantify nuclear immunoreactivity. A dynamic intensity range was used to classify cells as positive (above threshold) or negative (below threshold) while minimizing background noise.
5. Automated Cell Counting and Labeling Index Calculation: The proportion of ERα positive and ERβ positive cells was calculated relative to the total cell count in the analyzed region. The labeling index was expressed as the percentage of positively stained cells per field of view.

ERα and ERβ expression was assessed separately in TCL fibroblasts, FT fibroblasts, and FT synovial lining cells. Only cells exhibiting distinct nuclear immunostaining were classified as positive.

### 2.3. Statistical Analysis

Statistical analyses were performed using SAS (version 9.4, SAS Institute, Cary, NC, USA).

The normality of the data distribution was assessed using the Shapiro–Wilk test. Since not all variables adhered to a normal distribution, and the limited sample size was limited, non-parametric tests were selected to ensure methodological consistency. The Wilcoxon signed-rank test was used for paired comparisons, the Wilcoxon rank-sum test (Mann-Whitney U test) was applied for independent group comparisons, and Fisher’s exact test was employed for categorical binary variables.

The level of statistical significance was set at *p* < 0.05.

## 3. Results

### 3.1. Study Group

The patients’ mean age was 55.57 years ± 3.99, the mean interval of symptom onset–AI administration was 1.86 months ± 1.46, and AIA diseases (different from CTS) were concomitant in 71.43% of cases.

The mean value of sensitive velocity and motor latency in nerve conduction studies was 17.46 m/s ± 16.93 and 5.89 ms ± 2.18, respectively.

The Estrone and 17βestradiol values in serum were out of range in 28.57% and 16.67% of cases, respectively.

The preoperative and postoperative mean values of the Boston carpal tunnel syndrome questionnaire (BCTQ) were 62.57 ± 13.70 and 22.57 ± 6.27, respectively.

No surgical complications occurred. The data are summarized in Table 2.

#### 3.1.1. Intraoperative Appearance

Upon sectioning, TCL consistence was normal in 42.86% of cases, mildly stiff in 42.86%, and stiff in 14.28%.

FT thickening was mild (without adhesion) in 14.29% of cases and extensive (with adhesion) in 85.71%.

Nerve appearance was normal in 85.71% of cases, while a neuroma was detectable in 14.29%. The data are summarized in Table 3.

#### 3.1.2. Immunohistochemical Analysis (Figure 2 and Figure 3)

The mean value of ERα expression in TCL fibroblasts, FT fibroblasts, and FT synovial lining cells was 6.49% ± 3.84, 7.43% ± 5.41, and 8.11% ± 10.92, respectively.

The mean value of ERβ expression in TCL fibroblasts, FT fibroblasts, and FT synovial lining cells was 69.03% ± 25.55, 74.48% ± 29.55, and 92.49% ± 4.02, respectively. The data are summarized in Table 4.

### 3.2. Control Group

The mean age among patients was 55.43 ± 2.51 years, the mean interval of symptom onset–menopause was 3.14 months ± 2.12, and AIA-like diseases (different from CTS) were concomitant in 28.57% of cases.

The mean value of sensitive velocity and motor latency in the nerve conduction analyses was 25.43 m/s ± 17.78 and 6.90 ms ± 4.70, respectively.

The Estrone and 17βestradiol value in the serum was out of range in 50% and 16.67% of cases, respectively.

The preoperative and postoperative mean value of BCTQ was 69.71 ± 15.43 and 23.14 ± 3.58, respectively.

No surgical complications occurred. The data are summarized in Table 2.

#### 3.2.1. Intraoperative Appearance

Upon sectioning, the TCL consistency was mildly stiff in 42.86% and stiff in 57.14%.

FT thickening was extensive (with adhesion) in 100% of cases.

Nerve appearance was normal in 71.43% of cases, while a neuroma was detectable in 28.57%. The data are summarized in Table 3.

#### 3.2.2. Immunohistochemical Analysis (Figure 2 and Figure 3)

The mean value of ERα expression in TCL fibroblasts, FT fibroblasts, and the FT synovial lining cells was 4.81% ± 6.68, 14.14 ± 22.12 and 10.40% ± 19.29, respectively.

The mean value of ERβ expression in TCL fibroblasts, FT fibroblasts, and the FT synovial lining cells was 82.82% ± 17.98, 95.50% ± 8.38, and 96.45% ± 5.01, respectively. The data are summarized in Table 4.

## 4. Discussion

Estrogens have a wide range of effects on bone, muscle, and connective tissues [10,11,12,13,14]. Chondroprotective (proteoglycans increase and metalloproteinase-3 and nitric oxide reduction [15]), antinociceptive (kappa-opioid analgesic system mediated [16,17]), and anti-inflammatory (IL- 1β, TNF-α [18], and IL-6 reduction [19]) properties have been described. Estrogens also reduce fibroblast proliferation and collagen synthesis in ligaments [20,21], as well as collagen synthesis in the ligaments and fascia [22] and collagen synthesis in joint capsule [23]. Nevertheless, the hormone role is still controversial.

Estrogen signaling is complex. Estrogens exert their effects by interacting with ERs, members of the nuclear receptor superfamily. There are two subtypes of ERs, ERα and ERβ, ligand activated transcription factors that increase or decrease the transcription of target genes. ERs have different tissue distributions and transcriptional effects [24]; when coexpressed, ERβ can inhibit ERα mediated transcriptional activation [25].

In 2022, BC was found to be the leading cause of female malignant tumors [1]. About two-thirds of BC cases are positive for estrogen or progesterone receptors (or both). Hormone therapy—especially that using tamoxifen or AIs—is a mainstay of treatment for these patients [2].

Tamoxifen, a selective estrogen receptor modulator (SERM), is a competitive inhibitor of estrogens. Binding to ERs, tamoxifen exerts an antagonist effect in breast cancer along with an agonist response in several non-breast tissues, from which serious adverse effects such as endometrial cancer and thromboembolism may derive.

Other drugs have been developed to enhance tamoxifen’s efficacy and reduce its toxicity.

AIs reduce estrogen production by binding to aromatase. In premenopausal women, AIs also increase gonadotropin production, thus limiting the pharmacological suppression of estrogen levels.

According to their structure and mechanism of action, AIs are classified as first, second, or third generation and further subdivided into type 1 or 2. Those of type 1, steroidal AIs, irreversibly bind to aromatase hormone site. Those of type 2, nonsteroidal AIs, reversibly bind to the enzyme heme group.

Third generation AIs include exemestane (steroidal), anastrazole, and letrozole (nonsteroidal), with have similar efficacy and toxicity. First and second generation AIs are no longer used.

AIs reduce the aromatization of androgens to estrogens by more than 95% after 1 month of administration [26], reducing cell proliferation and increasing cell apoptosis in BC [27]. The main indication of AIs is as an adjuvant for postmenopausal women with early stage HR+ BC (eventually following prior tamoxifen) and as an adjuvant for premenopausal women (<35 years old or requiring chemotherapy) with early stage HR+ BC (combined with ovarian suppression). AIs are additionally used to treat postmenopausal women with advanced or metastatic HR + BC [26].

AIs present a more favorable risk–benefit profile than that of tamoxifen, with superior antitumor efficacy and lower life-threatening adverse effects [28].

Aromatase-inhibitor-induced arthralgia (AIA) refers to a set of musculoskeletal disorders that may occur in BC women treated with AIs. AIA includes arthralgia, carpal tunnel syndrome, tendinitis, stiffness, myalgia, and bone pain. Nevertheless, there is presently no consensus definition for AIA.

AIA is one of the leading causes of AI discontinuation, with non-compliance rates as high as 31% at one year (and up to 50% at 3 years), accompanied by a possible increase in cancer mortality [8].

The prevalence of AIA is 50% (range 20–70%) [29]. Symptoms usually appear within 2–3 months (with a range of 2 weeks–more than 10 months) from AI administration and peak at the sixth month [30]. If the drug is suspended, there is immediate relief of symptoms; on the contrary, recurrence occurs as soon as it is reintroduced [18].

The etiopathogenesis of this disorder is not understood but is likely related to estrogen deprivation.

Borrie and Kim have suggested that this disorder may be caused not only by low estrogen levels, but also by a sudden estrogen decline. These authors also reported numerous potentially implicated genic polymorphisms [31].

Tenti et al. highlighted the chondroprotective, antinociceptive, and anti-inflammatory properties of estrogen. The authors also reported potentially related risk factors and genic polymorphisms [27].

In a systematic review of the literature on AI-CTS [32], prevalence ranged from 0.8 [5] to 3.4% [6,7]. However, this value is considered to be underestimated due to the progressive increase in AI use and the lack of disease recognition. Although the reported prevalence is consistent with that in the general population (2.7 [33] to 5.8% [34]), this number increases to 26% within the first year of AI administration [35]. Drug discontinuation ranges from 2‰ [36] to 67% [5]. Chung et al. reported several risk factors: being aged 45 to 60 years old, having prior hormone replacement therapy [4,6,36], being treated with prior chemotherapy [4], having a body mass index greater than 25 kg/m [6,37], having prior musculoskeletal symptoms [6,36], and having a history of affective disorders [7].

Shin et al. [38] observed a bilateral involvement in 82.4% of AI-CTS patients.

CTS results from compression of the median nerve and is caused by any condition that increases pressure inside the carpal tunnel. FT thickening is often evident intraoperatively, especially if a rapid onset of symptoms has occurred [39].

CTS is considered idiopathic in most cases [39]. The most common histological finding is the subsynovial connective tissue noninflammatory fibrosis [40,41,42,43,44]. The main alterations are increases in fibroblast number and density, collagen fiber dimension, and collagen type III expression; vascular proliferation; and vascular hypertrophy with intimal thickening [44], indicating a modification of tissue mechanical properties.

CTS is more frequent in women than in men (65–75% of cases) [45]. Menopause [46], pregnancy [47], bilateral oophorectomy [48], oral contraceptives, and hormone replacement therapy [49] are some of the risk factors for CTS, suggesting a hormonal role (especially of estrogens) in the etiopathogenesis of this syndrome [46]. Despite the “hormone hypothesis,” few studies have investigated the expression of female sex hormone receptors in carpal tunnel tissues.

Toesca et al. [50] compared idiopathic CTS women with those lacking CTS by first demonstrating the presence of ERα and progesterone receptors (PRs) in TCL and synovial tissue. ERα was expressed in TCL fibroblasts and vascular walls, as well as in synovial tissue fibroblasts and lining cells. However, PR was expressed only in TCL fibroblasts and vascular walls.

Kim et al. [51] compared postmenopausal women suffering from idiopathic CTS with those lacking CTS. ERα and ERβ were expressed in FT fibroblasts, synovial lining cells, and vessel endothelial cells. Moreover, ERβ was more common than ERα.

Yamanaka et al. [52] evaluated ERα, ERβ, collagen type I A1 (Col1A1), collagen type III A1 (Col3A1), connective tissue growth factor (CTGF), and vascular endothelial growth factor (VEGF) expression in subsynovial connective tissue fibroblasts collected from postmenopausal women with idiopathic CTS. The authors concluded that ERα downregulates Col1A1 and Col3A1 expression, thereby reducing collagen type I and collagen type III synthesis. However, estradiol low concentration was unable to act on ERs.

Finally, Mohammadi et al. [53] compared postmenopausal women suffering from idiopathic CTS with those lacking CTS, but found no difference in TCL ER expression or serum estradiol levels.

Data from all the aforementioned studies are summarized in Table 5.

Estradiol serum levels drop severely in postmenopausal women [54,55]. At the same time, tissue ERα expression was found to peak in the group of 50–70 year old women with idiopathic CTS [51].

Previous studies have already demonstrated a greater representation of ERs in postmenopausal women with idiopathic CTS than in those without CTS [51] and in 50–70 year-old women with idiopathic CTS compared to other age groups of women and men with idiopathic CTS [51], thereby correlating an increase in the expression of ERs with a CTS form induced by estrogen deprivation.

The presence of ERs is essential for a direct effect of estrogens on a given tissue. The expression of ERs is both hormone- and cell-type-dependent [56,57]. Immunohistochemical staining in our study group revealed ERα and ERβ expression in AI-CTS tissues, making them a target of direct estrogen action.

Although comparable to the sample size in similar research [50,51,52,53], the small number of patients remains a limitation of our study. A small sample size can influence the power of statistical tests, increasing the possibility of a type II error (failure to detect significant differences between groups even in the presence of real differences). For this reason, a descriptive discussion of the results is provided in addition to their statistical analysis.

In both the study and control groups, ERβ was more commonly represented than ERα in TCL fibroblasts, FT fibroblasts, and FT synovial lining cells (*p* < 0.05). ERβ, therefore, plays a main role in AI-CTS and PI-CTS pathogenesis.

With the exception of ERβ in FT fibroblasts (more commonly expressed in the control than the study group, *p* < 0.05), there was no difference observed in the representation of TCL fibroblasts, FT fibroblasts, and FT synovial lining cell ERs between the study and control group. This result suggests, as in PI-CTS, an estrogen-related etiogenesis in AI-CTS. Differences in the expression of ERs—which were present but not statistically significant—are likely related to the shorter period of time elapsed in the symptom onset–AI administration interval compared to that in the symptom onset–menopause interval.

The postoperative BCTQ score was lower than the preoperative BCTQ score in both the study and control groups (*p* < 0.05), demonstrating the efficacy of surgical treatment in patients. Due to the frequency of extensive FT thickening, open carpal tunnel release with flexor tenosynoviectomy is recommended over minimally invasive techniques.

Conversely, there was no difference in preoperative and postoperative BCTQ scores between the study and control groups. The intensity of preoperative and residual symptoms perceived by patients was therefore the same, regardless of the cause of estrogen deprivation.

Moreover, there was no difference in nerve conduction study values (both sensitive velocity and motor latency) or serum hormone out-of-range values (both estrone and 17βestradiol) between the study and control groups.

The symptom onset–AI administration interval was shorter than the symptom onset–menopause interval. Additionally, concomitant AIA diseases are more frequent than concomitant AIA-like diseases. These differences, despite not being statistically significant, are likely related to the more rapid estrogen deprivation induced by AI administration compared to that induced by menopause.

Another limitation of our study is the absence of a control group composed of normal patients. Kim et al. [51] evaluated ER expression in the FT fibroblasts and FT synovial lining cells of postmenopausal idiopathic CTS women (study group) and postmenopausal women without CTS (control group), concluding that the ERα and ERβ labeling indexes were higher in the study than in the control group for both cell types (*p* < 0.001). Comparing our study group with the control group of and Kim et al., ERβ (likely the ER subtype with a main role in estrogen-related CTS pathogenesis) was more commonly represented in the FT fibroblasts and FT synovial lining cells of the study group (*p* < 0.05 and *p* < 0.01, respectively). Conversely, no difference in ERα expression was observed between the study and control groups. 

The statistical data are summarized in Table 6.

## 5. Conclusions

AI-CTS is a form of disease with a new definition, representing one of the main causes of AI discontinuation due to its negative impact on the patient’s quality of life.

Our study demonstrated the expression of ERs in AI-CTS tissues, correlating etiogenesis with estrogen deprivation.

Surgery is an effective treatment with low morbidity.

It is essentially important for clinicians to identify AI use as a possible risk factor for CTS development. Early diagnosis and prompt management can improve patient compliance and overall BC treatment outcomes.

## Figures and Tables

**Figure 1:**
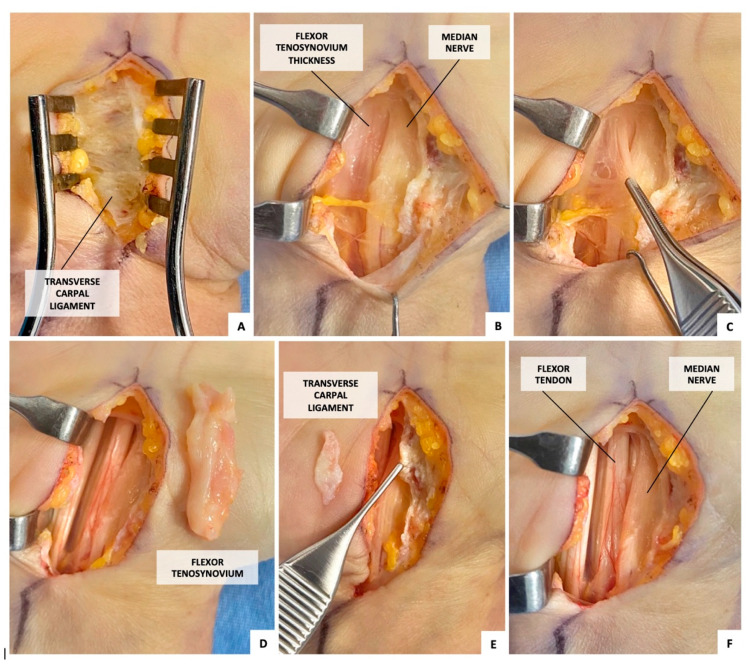
Surgical procedure. (**A**) transverse carpal ligament; (**B**) flexor tenosynovium and median nerve appearance after transverse carpal ligament sectioning; (**C**,**D**) flexor tenosynovium sample collection; (**E**) transverse carpal ligament sample collection; (**F**) flexor tendon and median nerve appearance after flexor tenosynoviectomy and median neurolysis.

**Figure 2:**
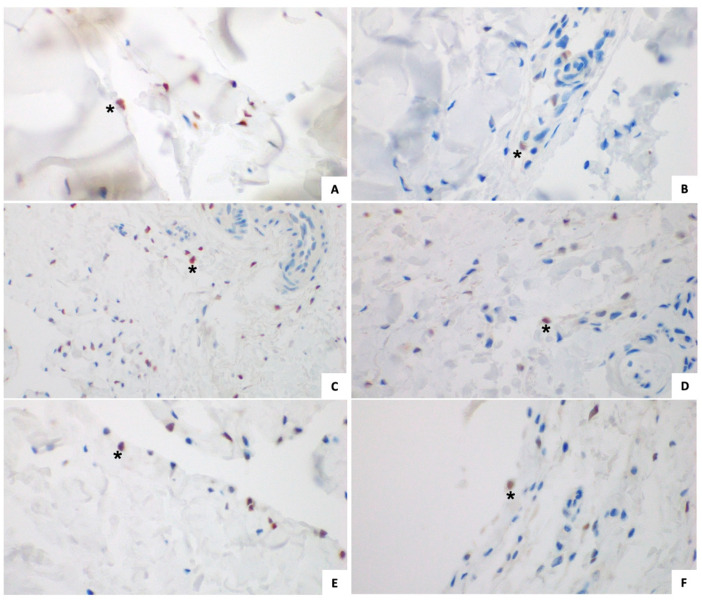
ERα expression. (*) example of an Erα-positive cell; (**A**) study group TCL fibroblasts; (**B**) control group TCL fibroblasts; (**C**) study group FT fibroblasts; (**D**) control group FT fibroblasts; (**E**) study group FT synovial lining cells; (**F**) control group FT synovial lining cells. ER, estrogen receptor; TCL, transverse carpal ligament; FT, flexor tenosynovium.

**Figure 3:**
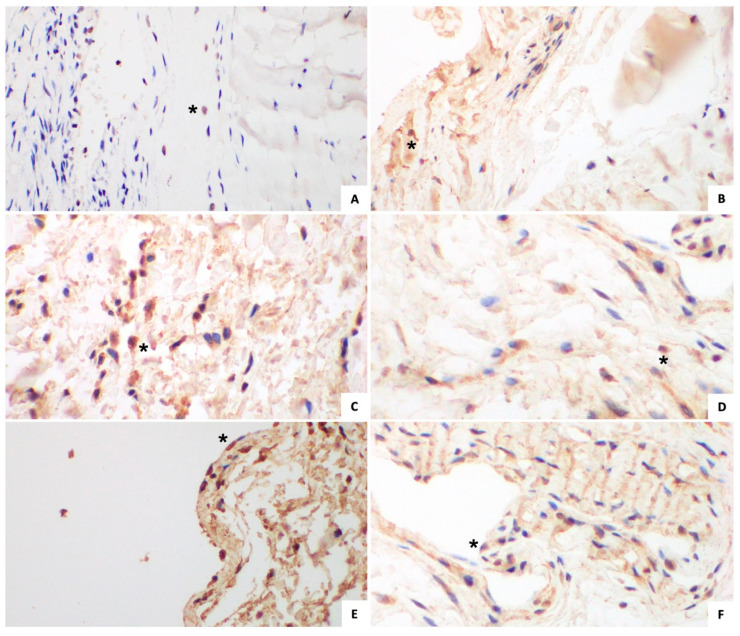
ERβ expression. (*) example of an Erβ-positive cell; (**A**) study group TCL fibroblasts; (**B**) control group TCL fibroblasts; (**C**) study group FT fibroblasts; (**D**) control group FT fibroblasts; (**E**) study group FT synovial lining cells; (**F**) control group FT synovial lining cells. ER, estrogen receptor; TCL, transverse carpal ligament; FT, flexor tenosynovium.

**Table 1:** Clinical data.

CASE	AGE AT SYMPTOM ONSET (yy)	HORMONE REPLACEMENT THERAPY (PRIOR)	SURGERY	LYMPH NODE (POSITIVE)	CHT	RDT	AROMATASE INHIBITOR	TAMOXIFEN	SYMPTOM ONSET - AI ADMINISTRATION (TIME ELAPSED, mm)	DOMINANT HAND	CONCOMITANT AIA DISEASES
AI-CTS 1	53	NO	Q, M	0, 1	YES	YES	LETROZOLE	YES	0	YES	NO
AI-CTS 2	56	NO	Q	1	NO	YES	LETROZOLE	NO	1	NO	YES
AI-CTS 3	60	NO	M	0	YES	YES	LETROZOLE	NO	3	YES	NO
AI-CTS 4	52	NO	M	0	YES	NO	EXEMESTANE	YES	1	YES	YES
AI-CTS 5	58	NO	Q	0	NO	YES	LETROZOLE	NO	3	NO	YES
AI-CTS 6	60	NO	Q	0	NO	YES	LETROZOLE	NO	4	NO	YES
AI-CTS 7	50	NO	M	2	YES	NO	EXEMESTANE	NO	1	YES	YES
									**SYMPTOM ONSET** **-** **MENOPAUSE** **(TIME ELAPSED, mm)**		**CONCOMITANT AIA-LIKE DISEASES**
PI-CTS 1	54	NO	-	-	-	-	-	-	6	YES	NO
PI-CTS 2	53	NO	-	-	-	-	-	-	3	NO	NO
PI-CTS 3	56	YES	-	-	-	-	-	-	1	NO	YES
PI-CTS 4	57	NO	-	-	-	-	-	-	1	YES	YES
PI-CTS 5	60	NO	-	-	-	-	-	-	6	YES	NO
PI-CTS 6	53	NO	-	-	-	-	-	-	3	YES	NO
PI-CTS 7	55	YES	-	-	-	-	-	-	2	YES	NO

AI-CTS, aromatase-inhibitor-induced carpal tunnel syndrome; PI-CTS, postmenopausal idiopathic carpal tunnel syndrome; CHT, chemotherapy; RDT, radiation therapy; AI, aromatase inhibitor; AIA, aromatase-inhibitor-induced arthralgia; Q, quadrantectomy; M, mastectomy; R, right; L, left; mm, months; yy, years.

**Table 2:** Instrumental, serum, and questionnaire data.

CASE	SENSORY NCS, VELOCITY (m/s, nv > 45)	MOTOR NCS, LATENCY (ms, nv < 4.0)	SERUM, ESTRONE (nv 30.92–99.82 pg/mL)	SERUM, 17BESTRADIOL (nv 0–66 pg/mL)	BCTQ, PREOPERATIVE (pv 19–95)	BCTQ, POSTOPERATIVE (pv 19–95)
AI-CTS 1	29.7	5.25	IN	IN	53	19
AI-CTS 2	0	5.17	IN	IN	62	34
AI-CTS 3	24.2	5.50	IN	IN	47	19
AI-CTS 4	29	5	OUT (↑)	IN	53	19
AI-CTS 5	0	10.5	IN	OUT (↓)	69	19
AI-CTS 6	0	6.2	IN	IN	66	29
AI-CTS 7	39.3	3.6	OUT (↑)	NULL	88	19
PI-CTS 1	38.7	4.4	IN	IN	50	19
PI-CTS 2	42.3	4.7	OUT (↓)	OUT (↓)	48	21
PI-CTS 3	33	4.5	IN	IN	77	19
PI-CTS 4	32	4.4	OUT (↑)	IN	86	27
PI-CTS 5	0	17.1	OUT (↑)	IN	79	27
PI-CTS 6	32	5.04	IN	IN	66	26
PI-CTS 7	0	8.19	NULL	NULL	82	23

AI-CTS, aromatase-inhibitor-induced carpal tunnel syndrome; PI-CTS, postmenopausal idiopathic carpal tunnel syndrome; NCS, nerve conduction study; BCTQ, Boston carpal tunnel syndrome questionnaire; IN, in range; OUT, out of range; (↑), increased; (↓), reduced; NULL, not assessable; nv, normal values; pv, possible values.

**Table 3:** Intraoperative appearance.

CASE	TCL (CONSISTENCE)	FT (THICKENING)	MEDIAN NERVE (APPEARANCE)
AI-CTS 1	NORMAL	EXTENSIVE	NORMAL
AI-CTS 2	MILDLY STIFF	EXTENSIVE	NORMAL
AI-CTS 3	MILDLY STIFF	EXTENSIVE	NEUROMA
AI-CTS 4	STIFF	EXTENSIVE	NORMAL
AI-CTS 5	MILDLY STIFF	MILD	NORMAL
AI-CTS 6	NORMAL	EXTENSIVE	NORMAL
AI-CTS 7	NORMAL	EXTENSIVE	NORMAL
PI-CTS 1	STIFF	EXTENSIVE	NORMAL
PI-CTS 2	MILDLY STIFF	EXTENSIVE	NORMAL
PI-CTS 3	STIFF	EXTENSIVE	NEUROMA
PI-CTS 4	STIFF	EXTENSIVE	NEUROMA
PI-CTS 5	MILDLY STIFF	EXTENSIVE	NORMAL
PI-CTS 6	STIFF	EXTENSIVE	NORMAL
PI-CTS 7	MILDLY STIFF	EXTENSIVE	NORMAL

AI-CTS, aromatase-inhibitor-induced carpal tunnel syndrome; PI-CTS, postmenopausal idiopathic carpal tunnel syndrome; TCL, transverse carpal ligament; FT, flexor tenosynovium.

**Table 4:** Immunohistochemical analysis (labeling index).

	TCL	TCL	FT	FT	FT	FT
CASE	FIBROBLASTS ERα	FIBROBLASTS ERβ	FIBROBLASTS ERα	FIBROBLASTS ERβ	SYNOVIAL LINING CELLS ERα	SYNOVIAL LINING CELLS ERβ
AI-CTS 1	6.40	15.46	7.48	94.09	8.15	96.72
AI-CTS 2	10.53	93.04	2.54	78.06	0.47	95.07
AI-CTS 3	4.20	75.23	1.12	9.05	2.54	92.76
AI-CTS 4	0.15	69.05	8.34	80.59	30.00	95.56
AI-CTS 5	9.43	89.74	12.54	93.57	5.43	85.23
AI-CTS 6	7.31	70.34	6.89	80.59	4.71	92.76
AI-CTS 7	8.34	70.34	14.61	85.43	6.21	89.34
PI-CTS 1	0.20	93.50	3.50	99.23	1.24	99.56
PI-CTS 2	0.00	91.34	63.09	97.04	53.43	95.61
PI-CTS 3	12.65	85.70	12.68	99.50	7.68	95.34
PI-CTS 4	16.14	83.45	12.41	98.30	8.45	99.45
PI-CTS 5	0.85	94.76	4.69	98.61	0.34	100.00
PI-CTS 6	2.61	87.85	0.07	99.26	0.34	99.20
PI-CTS 7	1.23	43.12	2.57	76.59	1.32	85.98

AI-CTS, aromatase-inhibitor-induced carpal tunnel syndrome; PI-CTS, postmenopausal idiopathic carpal tunnel syndrome; TCL, transverse carpal ligament; FT, flexor tenosynovium; ER, estrogen receptor.

**Table 5:** Studies on female sex hormone receptor expression in carpal tunnel tissues.

STUDY	TOESCA ET AL. [50]	KIM ET AL. [51]	MOHAMMADI ET AL. [53]	YAMANAKA ET AL. [52]
STUDY GROUP CONTROL GROUP	*idiopathic CTS* 23W, 7M *no CTS* 2W, 2M	*postmenopausal idiopathic CTS* 12W *postmenopausal no CTS* 6W	*postmenopausal idiopathic CTS* 12W *postmenopausal no CTS* 10W	*postmenopausal idiopathic CTS* 10W -
INVESTIGATIONS (TISSUES, CELLS, RECEPTORS, OTHER)	*TCL* fibroblasts *synovial tissue* fibroblasts, lining cells **ERα, PR**	*FT* fibroblasts, synovial lining cells **ERα, ERβ**	*TCL* fibroblasts **ER** *serum* estradiol	*subsynovial connective tissue* fibroblasts **ERα, ERβ, Col1A1, Col3A1, CTGF, VEGF**
MAIN CONCLUSIONS	ERα was expressed in TCL fibroblasts (and vascular walls) and in synovial tissue fibroblasts and lining cells PR was expressed in TCL fibroblasts (and vascular walls) ERα and PR were more commonly expressed in the study group than in the control group ERα and PR were more commonly expressed in the group of 50–70 year-old women with idiopathic CTS than in the other age groups of women with idiopathic CTS ERα and PR were more commonly expressed in the group of 50–70 year-old women with idiopathic CTS than in the group of men with idiopathic CTS Estrogen plays a larger role (compared to progesterone) in CTS etiopathogenesis TCL plays a larger role (compared to synovial tissue) as a tissue target of hormonal action	ERα and ERβ were expressed in fibroblasts and synovial lining cells (and vessel endothelial cells) ERβ was more commonly expressed than ERα ERα and ERβ were more commonly expressed in the fibroblasts and synovial lining cells of the study group than in the control group there was no correlation between ERα or ERβ and age, symptom duration, or symptom severity	there was no difference in ER expression between the study group and control group there was no difference in estrogen levels between the study group and control group there was no correlation between ER expression and electrodiagnostic parameters or the Boston score there was no correlation between estrogen levels and electrodiagnostic parameters or the Boston score	ERα downregulates Col1A1 and Col3A1 expression reducing collagen I and collagen III synthesis A low concentration of estradiol is unable to act on ERs There is no correlation between ERβ expression and collagen I or collagen III synthesis There is no correlation between the percentage of Erα and Erβ expression and collagen I or collagen III synthesis

CTS, carpal tunnel syndrome; TCL, transverse carpal ligament; FT, flexor tenosynovium; ER, estrogen receptor; PR, progesterone receptor; Col1A1, collagen type I A1; Col3A1, collagen type III A1; CTGF, connective tissue growth factor; VEGF, vascular endothelial growth factor; W, women; M, men.

**Table 6:** Statistical data.

	*p*-VALUE	R	EXACT CI 95% (HL)	OR	CI 95%
**STUDY GROUP**					
ERα TCL fibroblasts vs. ERα FT fibroblasts	0.6875				
ERα FT fibroblasts vs. ERα FT synovial lining cells	0.5625				
ERβ TCL fibroblasts vs. ERβ FT fibroblasts	0.5781				
ERβ FT fibroblasts vs. ERβ FT synovial lining cells	0.0781				
ERα TCL fibroblasts vs. ERβ TCL fibroblasts	0.0313 *				
ERα FT fibroblasts vs. ERβ FT fibroblasts	0.0313 *				
ERα FT synovial lining cells vs. ERβ FT synovial lining cells	0.0313 *				
BCTQ preoperative vs. BCTQ postoperative	0.0156 *				
**CONTROL GROUP**					
ERα TCL fibroblasts vs. ERα FT fibroblasts	0.3750				
ERα FT fibroblasts vs. ERα FT synovial lining cells	0.0313 *				
ERβ TCL fibroblasts vs. ERβ FT fibroblasts	0.0156 *				
ERβ FT fibroblasts vs. ERβ FT synovial lining cells	0.8125				
ERα TCL fibroblasts vs. ERβ TCL fibroblasts	0.0156 *				
ERα FT fibroblasts vs. ERβ FT fibroblasts	0.0156 *				
ERα FT synovial lining cells vs. ERβ FT synovial lining cells	0.0156 *				
BCTQ preoperative vs. BCTQ postoperative	0.0156 *				
**STUDY GROUP VS. CONTROL GROUP**					
ERα TCL fibroblasts	0.5203	0.178	(−6.71–8.58)		
ERβ TCL fibroblasts	0.1594	0.376	(−5.19–24.42)		
ERα FT fibroblasts	1.0000	0	(−11.56–9.04)		
ERβ FT fibroblasts	0.0213 *	0.615	(4.52–21.17)		
ERα FT synovial lining cells	0.7206	0.099	(−7.98–5.87)		
ERβ FT synovial lining cells	0.0550	0.513	(0.05–9.86)		
Symptom onset-AI administration interval vs. Symptom onset-menopause interval	0.2896	0.283	(−1.00–3.00)		
BCTQ preoperative	0.4812	0.188	(−11.00–26.00)		
BCTQ postoperative	0.4945	0.183	(−7.00–8.00)		
NCS sensitive velocity	0.2390	0.315	(−7.30–32.00)		
NCS motor latency	0.5224	0.171	(−1.70–3.19)		
AIA/AIA-like disease concomitance	0.1696			6.2500	(0.61–63.54)
estrone serum	0.5582			2.5000	(0.25–24.72)
17estradiol serum	0.8865			1.0000	(0.05–20.83)
**STUDY GROUP VS. KIM ET AL. CONTROL GROUP**					
ERα FT fibroblasts	0.2873	0.162	(−11.66–5.44)		
ERβ FT fibroblasts	0.0133 *	0.615	(−72.57–40.06)		
ERα FT synovial lining cells	0.1490	0.300	(−20.02–6.00)		
ERβ FT synovial lining cells	0.0017 **	0.813	(−84.76–67.56)		

R, effect size; EXACT CI (HL), exact confidence interval of the Hodges–Lehmann median difference; OR, odds ratio; CI, confidence interval; TCL, transverse carpal ligament; FT, flexor tenosynovium; vs, versus; ER, estrogen receptor; AI, aromatase inhibitor; BCTQ, Boston carpal tunnel questionnaire; NCS, nerve conduction study; AIA, aromatase-inhibitor-induced arthralgia; *, *p* < 0.05; **, *p* < 0.01.

## Data Availability

The original contributions presented in this study are included in the article. Further inquiries can be directed to the corresponding author.
